# Accuracy of Distance Recordings in Eight Positioning-Enabled Sport Watches: Instrument Validation Study

**DOI:** 10.2196/17118

**Published:** 2020-06-24

**Authors:** Rahel Gilgen-Ammann, Theresa Schweizer, Thomas Wyss

**Affiliations:** 1 Swiss Federal Institute of Sport Magglingen Magglingen/Macolin Switzerland

**Keywords:** geographic information systems, GPS measurement error, sports, geographic locations, monitoring physical training, movement analysis

## Abstract

**Background:**

Elite athletes and recreational runners rely on the accuracy of global navigation satellite system (GNSS)–enabled sport watches to monitor and regulate training activities. However, there is a lack of scientific evidence regarding the accuracy of such sport watches.

**Objective:**

The aim was to investigate the accuracy of the recorded distances obtained by eight commercially available sport watches by Apple, Coros, Garmin, Polar, and Suunto when assessed in different areas and at different speeds. Furthermore, potential parameters that affect the measurement quality were evaluated.

**Methods:**

Altogether, 3 × 12 measurements in urban, forest, and track and field areas were obtained while walking, running, and cycling under various outdoor conditions.

**Results:**

The selected reference distances ranged from 404.0 m to 4296.9 m. For all the measurement areas combined, the recorded systematic errors (±limits of agreements) ranged between 3.7 (±195.6) m and –101.0 (±231.3) m, and the mean absolute percentage errors ranged from 3.2% to 6.1%. Only the GNSS receivers from Polar showed overall errors <5%. Generally, the recorded distances were significantly underestimated (all *P* values <.04) and less accurate in the urban and forest areas, whereas they were overestimated but with good accuracy in 75% (6/8) of the sport watches in the track and field area. Furthermore, the data assessed during running showed significantly higher error rates in most devices compared with the walking and cycling activities.

**Conclusions:**

The recorded distances might be underestimated by up to 9%. However, the use of all investigated sport watches can be recommended, especially for distance recordings in open areas.

## Introduction

### Background

There are many wearable devices on the market, especially in the health and sports sectors, that can access global navigation satellite system (GNSS) information [1]. A world survey of fitness trends identified wearable technologies such as GNSS-enabled watches and activity trackers as a key trend in 2016, 2017, and 2019 [2]. Conventional GNSS-enabled sport watches are predominant for a diverse population of active runners of different fitness levels [3,4]. For example, the wearable technologies used by runners during a half-marathon and marathon competition were as follows: 44.7% (437/977) were represented by GNSS-enabled sport watches and 18.5% (181/977) by mobile phones with a combined app to track running performance. In comparison, the proportions of heart rate monitors (37/977, 3.8%), wristband activity trackers (27/977, 2.8%), and smart watches (14/977, 1.4%) were quite low during these competitions. Wiesner et al [4] revealed that within runners using wearable technologies, the most frequent parameters of interest were the distance covered (523/617, 84.8%), time (441/617, 71.5%), and average speed (412/617, 66.8%). In that study, 3 out of 4 participants stated that they always trusted the data. As the users rely on these data to guide their training or competition, monitor their training volume, or plan their exercises, knowledge about GNSS accuracy is of importance [5].

### Prior Work

In a systematic review of mobile apps to quantify aspects of physical activity, 20% (5/25) of the studies investigated the validity of mobile apps when measuring distance using GNSS information [6]. Mean percentage errors ranged between 2% and 10%. A systematic review of the validity of consumer-wearable activity trackers in 2015 revealed 22 studies [7]. However, only one study reported information on recorded distance but not using GNSS information. Recently, Pobiruchin and coworkers [3] investigated the recorded distance data obtained from different GNSS-enabled devices and brands during a half-marathon competition. They revealed small mean absolute errors of 0.12 km (0.6%) during the 21.1 km course. In the only study investigating GNSS-enabled sport watches, results from the validity of the recorded distances showed 0.8%, 1.2%, and 6.2% error rates on a straight path with open sky, an urban path, and a forest path, respectively [5]. However, further investigations are needed on recorded distances obtained in standardized settings to learn about different brands and products in the sport sector. Different sport watches should be investigated simultaneously in various real-world scenarios, both area-wise and speed-wise [8-10].

### Difficulties in the Global Navigation Satellite System

To better understand why there might be difficulties in the accurate assessment of distance traveled by GNSS-enabled devices, one must comprehend how such devices work and what the GNSS signal affects, and therefore, how this impacts the measurement quality. Four main satellite implementations exist: GPS (United States), Global Navigation Satellite System (GLONASS, Russia), Galileo (European Union), and BeiDou (China). The number of satellites for GPS, GLONASS, Galileo, and BeiDou are 31, 27, 22, and 19, respectively, which circle the Earth twice a day in a precise orbit at an altitude of approximately 20,000 km [11-15]. Each satellite transmits a unique right-hand polarized signal and orbital parameters that allow GNSS-enabled devices to decode and compute the precise location of the satellite. The GNSS receiver measures the distance to each satellite by the amount of time it takes to receive a transmitted signal to exactly locate the user’s position on Earth [15]. Several factors affect the signal transmitted between the satellites and the GNSS receiver such as bad signal acquisition, number of satellites, signal multipath, satellite geometry, and GNSS receiver clock errors [1,15-17]. Bad signal acquisition can happen if the user of a GNSS-enabled device disregards any of the manufacturers’ main instructions to achieve a high GNSS signal: staying outside, regularly synchronizing the watch to the mobile app or computer to download the latest satellite data (= assisted GPS data), updating the watch’s GNSS setting for whatever activity, or choosing GPS + GLONASS or Galileo. The more satellites a GNSS receiver can detect, the better the accuracy. To calculate one’s 2D position (latitude and longitude), a GNSS receiver must be locked on to the signal of at least three satellites. Therefore, the user should remain stationary with the watch facing up during signal acquisition. Furthermore, the signal multipath and satellite geometry affect the transmitted signal. A user may get position errors or no position readings at all when a signal is blocked. This can occur because a GNSS signal does not penetrate any solid constructions or water. In addition, the GNSS signal is reduced by dense vegetation or cloudy weather or near objects and buildings, as there are reflections that transform the right-hand polarization into left-hand polarization before it reaches the GNSS receiver. Generally, the satellite signals are more effective when the satellites are located at wide angles relative to one another. Therefore, during signal acquisition, the user should stay away from large buildings and dense vegetation and, ideally, remain in a flat open area. Last, measurement quality can be hampered by timing errors the GNSS receiver might have because it is less accurate than the atomic clocks on GNSS satellites. The user, however, cannot change the clock errors in the GNSS receiver. In the northern hemisphere, the ground stations’ determined locations can vary due to the mentioned error sources. With GPS, GLONASS, and GPS + GLONASS, the determined horizontal (and vertical) location errors can be 8.0 (SD 17.1) m, 9.4 (SD 18.3) m, and 7.1 (SD 14.0) m, respectively, with a 95% confidence interval [18-20]. Overall, little to no information about positioning accuracy is provided by the most common manufacturers of GNSS-enabled sport watches. Scott et al [21] rated measures of validity of GPS in team sports as good (<5%), moderate (5% to 10%), or poor (>10%).

### Aims of the Study

The aim was to investigate the accuracy of and parameters affecting the recorded distances obtained by eight sport watches from Apple, Coros, Garmin, Polar, and Suunto when assessed in different areas and at different speeds under different external circumstances.

## Methods

### Study Setting

**Figure 1:**
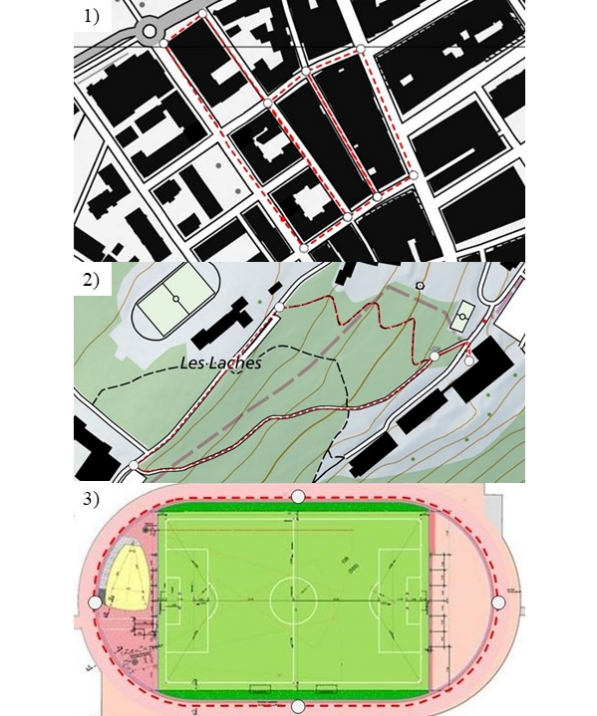
The urban (1), forest (2), and track and field (3) measurement areas. White circles divide the courses into subsections that were randomly combined and added up to result in different selected reference distances within the same setting.

In this instrument validation study, measurements were conducted in three areas while performing three different speed categories [10] (Figure 1):

- Urban area: in the city center of Biel/Bienne (Switzerland) at 434 m above sea level on a flat street with narrow and partly high buildings
- Forest area: in terrain in Magglingen (Switzerland) at 905 m above sea level on uphill (total gradient of 52 altitude meters and 11% slope), downhill, and flat paths with partly tall trees
- Track and field area: in an open track and field stadium in Magglingen without a tribune at 954 m above sea level on a 400 m track in the middle of lane 1 without any satellite visibility constraints

The courses in each area were marked to allow ideal replications of the measurements. All courses in a respective area were split into subsections that were randomly combined within one measurement area resulting in selected reference distances of 400 m to 4500 m. This was chosen to ensure variance of traveled distances as a possible independent variable within the same controlled setting and investigate whether the distance per se and number of travel direction changes affected the results. Subsections of each course were accomplished partly or entirely or repeated in either direction (including U-turns), or any combination of these (Figure 1). A trundle wheel [22] was used as the reference measure for all selected subsections. Each subsection was assessed twice with the trundle wheel with an accuracy to 1 cm.

Measurements were taken in the three speed categories—walking, running, and cycling—to represent low-, moderate-, and high-gait speeds [1]. These three speed categories were self-paced by the subject but steady—according to the subjective feeling—within one measurement. The cycling was performed using an electric bike (e-bike) [23] to ensure high speeds and steady and straight riding, particularly on the uphill section in the forest area. Self-pacing and different subsections were chosen to ensure data acquisition that represented different real-life situations. The activity task itself and steady speed were secondary, as the primary aim was to validate recorded distances. Also, having a range of reference distances allowed statistical analyses with normally distributed data.

### Instruments

Eight watches from the most common manufacturers in the field of sport watches (as of January 2019) were included in this study. The specific types were chosen based on personal communications with exercise physiologists and endurance athletes (see Table 1). All units were configured to the lowest possible 1-second (1 Hertz) GNSS recording, and the GPS + GLONASS satellite system was selected except for the Apple Watch Series 4 (Apple Inc), which does not have the option to choose the satellite system, and the V800 (Polar Electro Oy), which only has GPS due to its antenna implementation.

**Table 1:** Investigated sport watches and their specifications.

Sport watch model	Abbreviation	Manufacturer	Serial number	Firmware or OS version
Apple Watch Series 4	AW4	Apple Inc	FH7XD28MKDH9	5.2
Apex 46 mm	CoA	Coros Wearables Inc	73F855	1.31
Fenix 5X Plus	G5X+	Garmin Ltd	5MM005560	6.0 (7.6)^a^
Forerunner 935	GF935	Garmin Ltd	50S007800	12.50 (13.00)^a^
Vantage M	VM	Polar Electro Oy	464F832E	4.0.0
Vantage V	VV	Polar Electro Oy	4AF6F824	4.0.0
V800	V800	Polar Electro Oy	306AE719	1.11.49
Suunto 9 Baro	S9B	Suunto Oy	1.8251E+11	2.5.18 (2.6.54)^a^

^a^Updates were needed during data collection to synchronize data to the respective website. Firmware version is listed after the update.

### Subject

One healthy, fit, and lean person (female, aged 26 years, 53.0 kg, 1.58 m) performed all measurements. Having one subject ensured perfect standardized measurements. Moreover, independent variables such as body height, arm length and movement, walking pattern, etc, could be precluded. The subject was well familiar with the handling of all eight investigated watches and with the study design.

### Data Collection

Measurements were scheduled for different days (7:00 am to 6:00 pm) from April to July 2019. On measurement days, all sport watches were synchronized to the respective mobile app and computer software by the supervisor to download the latest satellite data.

**Figure 2:**
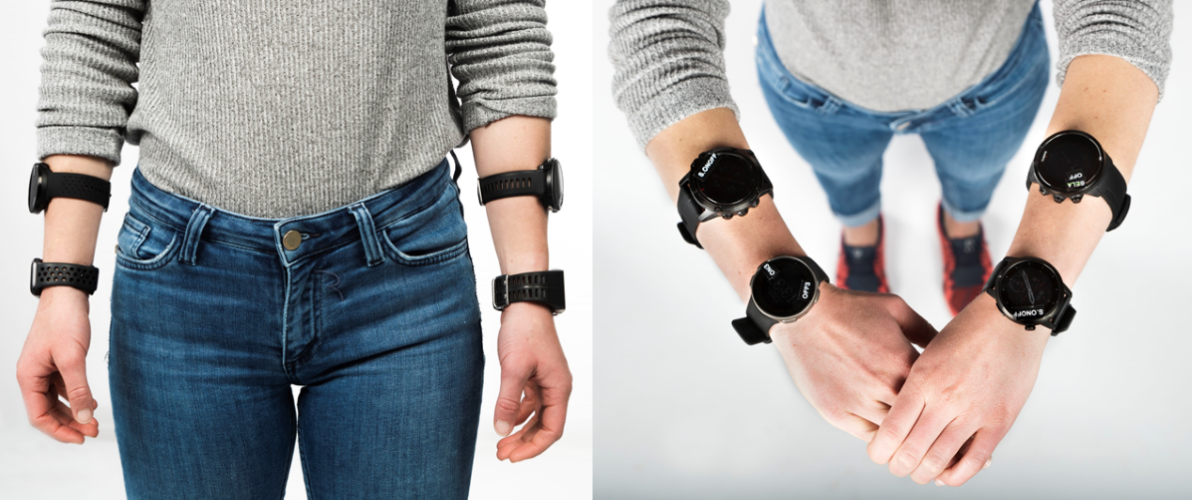
Randomly assigned wearing position on the forearm of the sport watches during one measurement (left). Calibration posture with arms outstretched to achieve the best global navigation satellite signal quality (right).

During each measurement, four watches were worn simultaneously. The subject wore two watches per forearm at least 4 cm apart to minimize potential interference between the devices (Figure 2). The combination of which four watches, the wearing side (wearing the watches on the right or left arm), and the wearing position (wearing the watch higher or lower on the forearm) were randomly assigned by the supervisor using a covariate adaptive randomization approach prior to the measurements [10,24]. It is worth noticing that the higher the watch is located on the arm, the stronger the signal-blocking effect due to the body’s interference. The watches were always mounted on bare skin and were not covered by sleeves. The subject prepared all the watches to receive the GNSS signal while standing in a flat, mostly open area without large buildings or dense vegetation. After readying each watch’s positioning connection, the subject waited another 5 minutes with arms outstretched, watches facing up, for calibration purposes to reach the best GNSS signal quality prior to starting the data acquisition (Figure 2).

Thereafter, the subject started the watches from left to right and did the same to stop the measurement. As the data collection was performed with one subject but eight watches, the same measurement—speed category and area with its combination of course subsections to reach the same reference distance lengths—was accomplished twice in a row, each time with four watches. Additionally, the following parameters were protocolled by the supervisor for each assessment: course turns per 1000 m, time of day, temperature, precipitation, sun, humidity, solar irradiance, and wind velocity [10]. In total, each of the three areas was completed four times in each of the three speed categories, resulting in 3 × 4 × 3 = 36 measurements.

### Data Processing

After each measurement, the data were uploaded to the respective software provided by and exported as default by the five investigated manufacturers. In the Garmin and Suunto devices, a firmware update was required during data collection to synchronize the data to the respective website. These data were not treated differently. To calculate the recorded distances of each watch, only the values of the real measurement time period were computed, except the data from the AW4, which could not be exported. In this case, the distance values shown on the AW4 display were noted, entered, and double checked in an Excel Windows 2016 (Microsoft Corporation) file by the supervisor for each measurement.

### Statistical Analysis

Descriptive statistics with mean absolute and percentage errors, dependent samples 2-tailed *t* tests, Bland-Altman analyses, and a ±5% accuracy of the recorded distance were used. The dependent *t* test was applied to test whether the difference of the recorded distances between tested devices and the reference values was zero. Bland-Altman analyses with corresponding 95% limits of agreement (SD 1.96) were used to calculate systematic errors in the recorded distances. The ±5% accuracy of the recorded distances was defined as the percentage at which the respective watch recording was within the proposed equivalence zone of ±5% from the reference values [21,25]. Furthermore, multivariate linear regression analyses with stepwise backward elimination were used for each watch to detect independent variables with significant influence on the mean absolute error (MAE). The independent predictor variables investigated were speed categories, area, time of day, temperature, turns per 1000 m, precipitation, sun, humidity, solar irradiance, wind velocity, watch wearing side (0=right arm; 1=left arm), and watch-wearing position (0=higher; 1=lower on the forearm). These were chosen as potential predictors, as they occur during a user’s everyday life (where to move, at what pace, how curvy the terrain is, etc); the wearing position on the higher and lower forearm was included to demonstrate whether the setup in the study affected the result. The adjusted *R*^2^ and β^2^ were used to estimate the explained variances of the dependent variable by all the included variables and by each independent variable, respectively. In the case of multicollinearity (*r*≥.80) or the nonsignificant prediction of the MAE, the relevant variable was excluded from the regression analysis. Any *P*<.05 was considered statistically significant, and the α level was .05. The statistical analyses were applied using SPSS Statistics 25.0 (IBM Corporation) and Excel.

## Results

### Main Findings

In total, 100% (36/36) of the measurements were recorded for each sport watch but in the S9B, 97% (35/36) of the measurements were analyzed due to a technical failure during one assessment in the forest area. The walking, running, and biking was accomplished on average at 5.4 (SD 0.2), 10.2 (SD 0.7), and 17.6 (SD 2.6) km/h, respectively.

**Figure 3:**
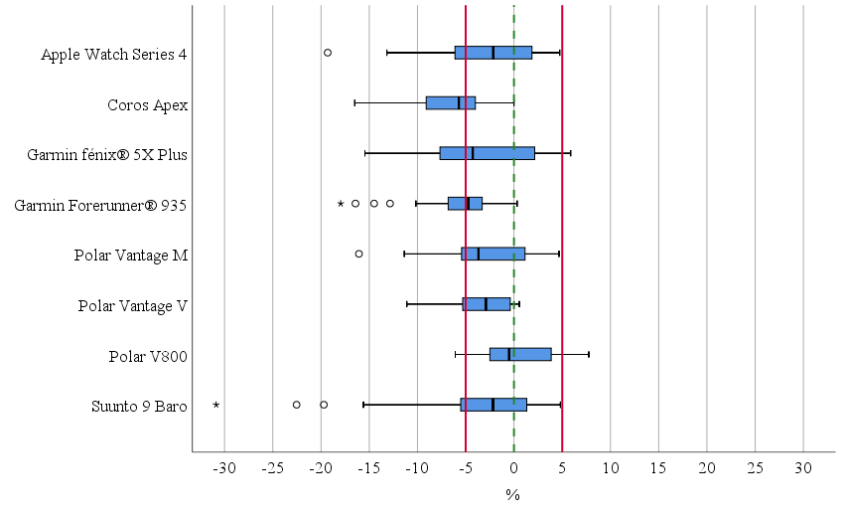
Relative deviation of the distances recorded by the 8 watches compared with the reference distance. The red lines indicate the proposed equivalence zone (±5% of the mean); the boxplots’ lower and upper boundaries indicate the 25% and 75% quantiles of the distance data, respectively, and the middle notch indicates the median data value. The whiskers include all data points that fall within the 1.5 interquartile range of the 25% and 75% quantile values. Circles and stars indicate distance data points that lie beyond the 1.5 and 3 interquartile ranges, respectively.

For all three measurement areas combined, the recorded systematic errors (limits of agreements) ranged between 3.7 (±195.6) m and –101.0 (±231.3) m for the V800 and CoA, respectively (Multimedia Appendix 1). The mean absolute percentage error (MAPE) ranged from 3.2% to 6.1% for the V800 and the S9B, respectively. Only the three GNSS receivers from Polar showed overall MAPEs <5%. On average, the mean recorded distances within ±5%, when compared with the reference values, ranged from 80.6% (29/36) in the V800 to 44.4% (16/36) in the G5X+ (Figure 3). Overall, only the AW4 (*P*=.08) and the V800 (*P*=.83) showed no statistically significant differences from the reference distance.

### Measurement Areas

Specifically, the recorded distances were significantly different from the measured distances in the forest (all *P*<.04), urban (all *P*<.03), and track and field areas (AW4, G5X+, and S9 all *P*<.001; Tables 2-4). The Bland-Altman analyses showed an underestimation by all watches in the forest and urban areas (except the overestimation in the V800) but an overestimation in the track and field area. Further, in all watches, the lowest MAE and good ±5% accuracy were recorded in the track and field measurements.

**Table 2:** Recorded distances and error rates of the eight sport watches obtained in the urban area when compared with the mean reference distance of 2046.4 (SD 1159.7) m (n=12).

Watch	Recorded distance (m), mean (SD)	*P* value	Systematic errors (m), (limits of agreement)	Mean absolute error (m), (mean absolute percentage error)	5% accuracy^a^, n (%)
AW4	1951.6 (1088.3)	.03	–94.8 (257.6)	108.4 (5.1)	7 (58)
CoA	1899.5 (1085.6)	.001	–146.9 (237.4)	146.9 (7.5)	4 (33)
G5X+	1939.9 (1114.9)	.003	–106.4 (186.4)	110.8 (5.9)	6 (50)
GF935	1857.1 (1054.5)	.003	–189.3 (344.7)	189.3 (8.9)	4 (33)
VM	1949.2 (1110.1)	.02	–97.9 (234.8)	109.8 (5.3)	7 (58)
VV	1941.9 (1118.9)	0	–104.5 (171.6)	105.3 (5.4)	5 (42)
V800	2134.6 (1227.1)	.003	88.2 (154.8)	89.4 (3.9)	8 (67)
S9B	1868.7 (1006.9)	.02	–177.7 (438.7)	191.6 (8.5)	5 (42)

^a^Percentage at which the distance recorded by each device was within 5% of the reference distance.

**Table 3:** Recorded distances and error rates of the eight sport watches obtained in the forest area when compared with the mean reference distance of 2111.6 (SD 1109.9) m (n=12).

Watch	Recorded distance (m), mean (SD)	*P* value	Systematic errors (m), (limits of agreement)	Mean absolute error (m), (mean absolute percentage error)	5% accuracy^a^, n (%)
AW4	1969.8 (1031.9)	.008	–141.8 (297.8)	148.3 (6.9)	5 (41.7)
CoA	1944.1 (1032.6)	<.001	–167.4 (177.2)	167.4 (8.5)	2 (16.7)
G5X+	1946.1 (1029.2)	<.001	–165.4 (175.7)	165.4 (8.2)	0 (0.0)
GF935	1983.0 (1037.5)	<.001	–128.6 (171.3)	128.6 (6.0)	4 (33.3)
VM	1993.4 (1048.7)	<.001	–118.2 (149.7)	118.1 (5.6)	6 (50.0)
VV	2000.4 (1047.4)	.001	–111.2 (157.8)	111.1 (5.0)	7 (58.3)
V800	2030.6 (1063.3)	.002	–81.0 (140.3)	81.0 (3.5)	9 (75.0)
S9B	1827.9 (988.1)	.07	–166.5 (529.4)	166.5 (7.5)	6 (54.5)^b^

^a^Percentage at which the distance recorded by each device was within 5% of the reference distance.

^b^n=11.

**Table 4:** Recorded distances and error rates of the eight sport watches obtained in the track and field area when compared with the reference distance of 2104.3 (SD 1167.4) m (n=12).

Watch	Recorded distance (m), mean (SD)	*P* value	Systematic errors (m), (limits of agreement)	Mean absolute error (m), (mean absolute percentage error)	5% accuracy^a^, n (%)
AW4	2196.4 (1227.4)	.001	92.1 (137.6)	92.1 (4.1)	8 (67)
CoA	2115.7 (1180.4)	.13	11.4 (47.3)	18.7 (0.9)	12 (100)
G5X+	2165.4 (1190.1)	.001	61.1 (86.7)	61.1 (3.0)	10 (83)
GF935	2121.6 (1182.2)	.20	17.2 (86.3)	31.2 (1.3)	12 (100)
VM	2142.3 (1214.5)	.049	38.0 (116.8)	48.7 (2.1)	12 (100)
VV	2134.1 (1206.0)	.05	29.8 (92.7)	43.8 (2.3)	10 (83)
V800	2108.2 (1171.5)	.85	3.9 (134.6)	53.4 (2.3)	12 (100)
S9B	2150.5 (1194.8)	.001	46.1 (65.4)	49.2 (2.5)	12 (100)

^a^Percentage at which the distance recorded by each device was within 5% of the reference distance.

### Affecting Parameters

The backward multiple linear regression analyses on each watch revealed different significant predictors of an increased MAE (Table 5). The included independent variables explained between 18.3% of the variance in the MAE in the AW4 and 44.2% in the CoA. The running category was the most shown predictor; in six watches, it remained and had a significant influence on the final regression models and explained between <1% and 9% of the MAE in the respective watches.

**Table 5:** Linear regressions for each device separately with the mean absolute error as dependent variable.

Watch and predictors		Interpretation^a^	Odds ratio (*P* value)	*R* ^2^	*F* value	Explained variance, β^2^ (%)
**AW**				.18	3.71	
	Running	Running over walking and cycling	76.48 (.05)			8
	Arm position	Lower forearm position	65.10 (.08)			10
**CoA**				.44	13.08	
	Track and field	Urban and forest over track and field	–138.47 (<.001)			37
	Running	Running over walking and cycling	59.49 (.05)			7
**G5X+**				.32	5.09	
	Track and field	Urban and forest over track and field	–104.34 (.002)			18
	Running	Running over walking and cycling	49.65 (.07)			8
	Forest	Forest over urban	-54.60 (.08)			<1
**GF935**				.40	7.14	
	Temperature	Lower temperatures	–14.11 (.002)			20
	Urban	Urban over forest and track and field	102.72 (.01)			16
	Time of day	Later in the day	394.14 (.02)			<1
**VM**				.21	4.49	
	Precipitation	More rain	39.15 (.03)			9
	Running	Running over walking and cycling	62.92 (.03)			9
**VV**				.43	4.54	
	Precipitation	More rain	47.19 (.003)			12
	Running	Running over walking and cycling	66.99 (.02)			6
	Solar irradiance	More solar irradiance	.11 (.05)			4
	Turns per 1000 m	More turns	5.73 (.05)			5
	Cycle	Cycling over walking	48.94 (.07)			1
**V800**				.39	6.91	
	Precipitation	More rain	43.53 (.001)			28
	Cycle	Cycling over running and walking	49.59 (.03)			8
	Running	Running over walking	42.39 (.06)			<1
**S9**				.27	3.79	
	Temperature	Lower temperatures	–24.25 (.004)			10
	Humidity	Less humid	–5.82 (.02)			2
	Sun	More clouds	–2.73 (.08)			1

^a^The mean absolute error was increased by the respective predictors.

## Discussion

### Principal Findings

The aim of this study was to evaluate the accuracy of the recorded distances of eight sport watches under different real-world environmental conditions for various speeds and reveal the predictors affecting measurement quality. Our results showed that the V800 was the most accurate watch overall, with a systematic error of 3.7 m, a MAPE of 3.2%, and 80.6% of all distance recordings within ±5% of the reference values. Notably, the V800 can use only GPS satellites due to its antenna implementation. Consequently, it is questionable whether the number of satellites and combination of different GPS + GLONASS or Galileo affects measurement quality that much, which was previously questioned [1]. In contrast, other devices showed a significant systematic error of up to –101.0 m and limits of agreements of over ±400 m, overall MAPEs of up to 6.1%, and less than 50% of the data falling within the tolerable range of ±5% (Figure 3). Overall, the recorded distances were underestimated in all watches, and the variance and some outliers were rather high. In contrast, during the Trollinger Half-Marathon, an overall MAPE of 0.6% was observed in the GNSS-enabled devices, of which the Garmin devices performed the most accurately [3]. In addition, the recorded distances generally showed an overestimation of the half-marathon distance. However, comparability with our study is limited, as our data were assessed under standardized conditions, whereas during the Trollinger Half-Marathon, all runners started/stopped and calibrated their devices individually and potentially did not run on the ideal route to complete the entire 21.1 km.

### Measurement Areas

Considering the different measurement areas separately, an underestimation of the recorded distances in the forest and urban areas (except for the V800) was observed. In these areas, the MAPEs ranged from –3.5% to –8.9%, and a low 5% accuracy of 0% to 75% indicated large variances. This was in line with the previous research, demonstrating an underestimation of the recorded distances by –1.2% and –6.2% in an urban and forest area, respectively [5]. These results underline the fact that the GNSS signal is reduced in obstructed conditions such as dense vegetation or near objects and buildings, as it may reflect off before it reaches the GNSS receiver (ie, the GNSS not only receives signals directly from the satellites, signals are also reflected off such surfaces) [16]. In contrast, in the track and field area, the recorded distances were overestimated compared with the reference distance. However, the MAPEs were all <5% and ranged from 0.9% to 4.1% only. Furthermore, a good 5% accuracy was shown in the track and field area, with 5 devices having 100% (CoA, GF935, VM, V800, S9B), 2 devices having 83% (G5X+, VV), and 1 device having 67% (AW4) of the distance recordings falling within the ±5% accuracy threshold. The authors assume that manufacturers may autocorrect the recorded distances in the first place to level out the underestimation in difficult areas, which may in turn result in an overestimation of the distance recordings in unobstructed conditions, such as flat and open areas [17].

### Affecting Parameters

The included independent variables explained as much as from 18.3% to 44.2% of the variance in the MAEs of the distance recordings. Additionally, the running category showed in 75% (6/8) of the sport watches significantly increased error rates in recorded distances when compared with walking and cycling speeds. We assume this error is related to the gait-induced arm swing than to the movement speed itself. In comparison, cycling was the activity with the highest absolute speed, but in 25% (2/8) of the watches, it remained in the final regression model only. Previously, research compared the recorded altitude gains when assessed in the same brand on sport watches simultaneously placed on the wrist and on the hip when walking or running [26]. The watch placed on the hip was always more accurate than the watch placed on the wrist, and the error in altitude measures increased with faster movement speed. It was argued that the gait-related arm swing negatively affected the measurement accuracy which, in running, is raised in amplitude and frequency compared with walking. Furthermore, in our study, more rain was a significant predictor of an increased MAE in 38% (3/8) of the watches, which might be related to the impaired measurement accuracy in cloudy weather.

### Practical Implications

Recent research highlighted the broad use of GNSS-enabled watches in runners of different fitness levels and that users trust the data of such devices [3,4]. However, our study showed, depending on what device was applied, that from 80.6% (29/36) to as little as 44.4% (16/36) of the mean recorded distances fell within ±5% when compared with the reference values. In particular, running over walking and cycling activities were shown to impair the GNSS accuracy in the recorded distances. Nevertheless, the use of all the investigated sport watches can be recommended, especially for distance recordings in an open area. Yet in case of training monitoring and regulation based on recorded distance data, one must be aware that the recorded distances might be underestimated by up to 9%. As such, correct execution of the manufacturers’ instructions is essential to get the best accuracy (ie, for the latest satellite data to be valid).

### Limitations

Although we controlled for the wearing side and wearing position of the sport watches, we cannot exclude potential interference between the devices [10]. Only 14% (5/36) of the measurements were accomplished with moderate to heavy precipitation. In addition, the independent variables watch-wearing side and watch-wearing position occurred in a limited number of measurements only. Therefore, the power in the regression analysis is reduced, which in turn diminishes the interpretation of these predictors of increased error rates. Our data acquisition was performed by a single subject to ascertain perfect standardization. However, we cannot exclude that a study sample with different anthropometrics would fully affirm our results. Last, the selection of the specific eight sport watches might be biased as it was based on personal communications with exercise physiologists and endurance athletes rather than based on a detailed market research.

### Conclusions

Our results showed that there was an overall moderate to good GNSS accuracy regarding recorded distances, with MAPEs ranging from 3.2% to 6.1% when assessed in urban, forest, and track and field areas. However, only three of the eight investigated GNSS-enabled sport watches reported an average MAPE <5%. Noticeably, in the unobstructed conditions of an open area, 75% (6/8) of the sport watches were able to accurately record distances, whereas in the obstructed conditions of forest and urban areas, this accuracy was limited, with a general underestimation of the covered distances. Furthermore, the data assessed during running showed significantly higher error rates in most devices compared with the walking and cycling activities.

## Appendix

Multimedia Appendix 1 Bland-Altman plots for each device distinguished by measurement areas and speed categories.

## Abbreviations

AW4: Apple Watch Series 4 (Apple Inc)
CoA: Apex 46 mm (Coros Wearables Inc)
G5X+: Fenix 5X Plus (Garmin Ltd)
GF935: Forerunner 935 (Garmin Ltd)
GLONASS: Global Navigation Satellite System
GNSS: global navigation satellite system
MAE: mean absolute error
MAPE: mean absolute percentage error
S9B: Suunto 9 Baro (Suunto Oy)
V800: V800 (Polar Electro Oy)
VM: Vantage M (Polar Electro Oy)
VV: Vantage V (Polar Electro Oy)
