# Supplementary figures and images for: Accuracy of Distance Recordings in Eight Positioning-Enabled Sport Watches: Instrument Validation Study
Source: JMIR Mhealth Uhealth. 2020 Jun 24;8(6):e17118. doi: 10.2196/17118 (PMC7381051; doi:10.2196/17118)

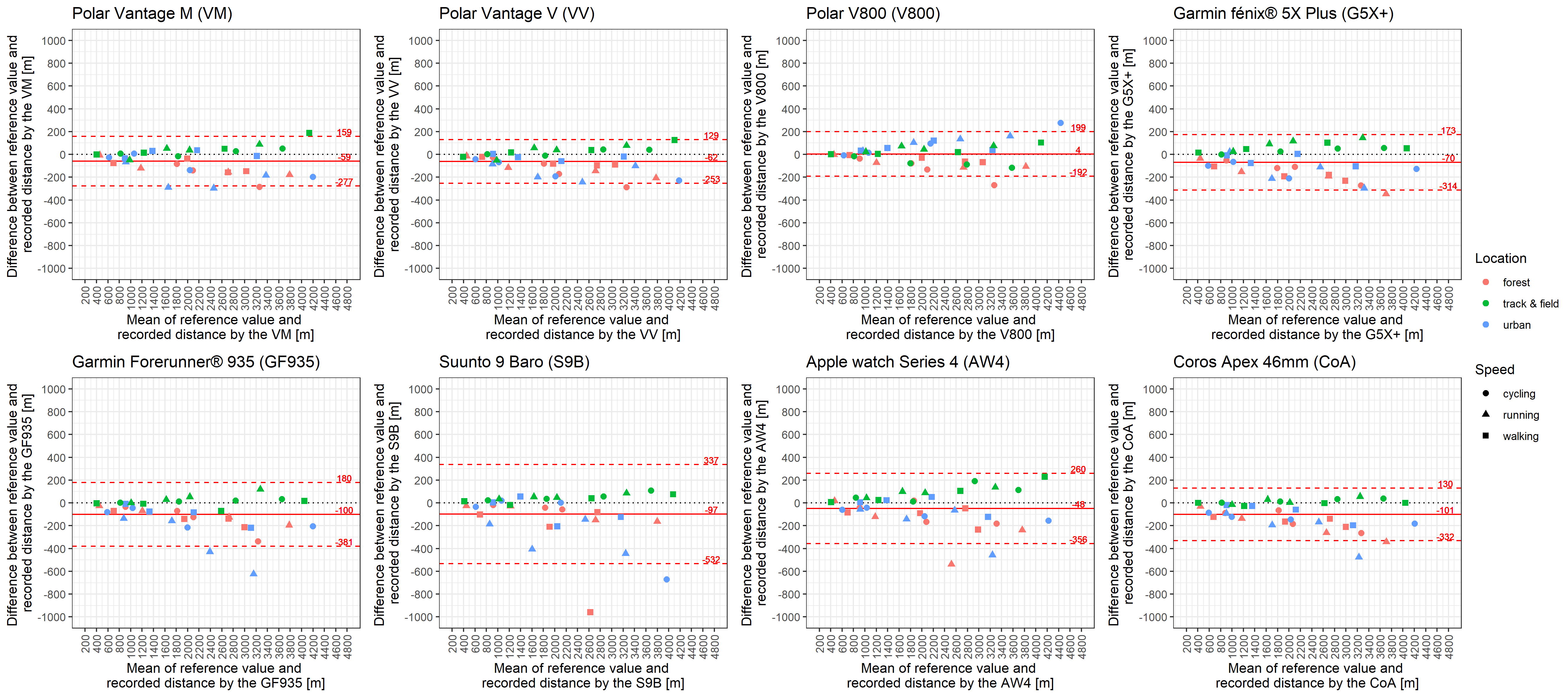

Supplement: Multimedia Appendix 1 [file mhealth_v8i6e17118_app1.png]
